# From “Omics” to Field: Deciphering the Stress Adaptation Networks and Breeding Potential of *Medicago ruthenica* L.

**DOI:** 10.3390/cimb48040365

**Published:** 2026-04-01

**Authors:** Chen Zhang, Yingfang Shen, Leping Qi, Xinxin Sun

**Affiliations:** 1 College of Ecology Environment and Resources, Qinghai Minzu University, Xining 810007, China; zhangc0615@163.com (C.Z.); qlplyc@163.com (L.Q.); sunxinxin2002@126.com (X.S.); 2 Key Laboratory of Resources Chemistry and Ecological Environment Protection on the Qinghai-Tibetan Plateau, State Ethnic Affairs Commission, Xining 810007, China; 3 Key Laboratory of High-Value Utilization of Characteristic Economic Plants in Qinghai Province, Xining 810007, China

**Keywords:** *Medicago ruthenica* L., leguminous forage, stress response, research hotspots

## Abstract

*Medicago ruthenica* L., a superior forage crop within the genus *Medicago* (Fabaceae), is endowed with remarkable stress tolerance and an abundance of bioactive compounds, conferring significant ecological and forage value. Existing reviews primarily focus on a single research direction, and the most recent findings are dated, failing to cover breakthroughs at the molecular level. This paper systematically synthesizes the latest research progress in five key areas: genetic diversity and genomic studies, biotic stress responses, abiotic stress tolerance mechanisms (drought, salinity, and low temperature, etc.), utilization (including genetic breeding, ecological restoration, and forage development), and future research prospects. This review addresses critical gaps in existing literature, particularly regarding advances in genomic sequencing, biotic stresses, and research on stress-associated microorganisms. Research indicates that *M. ruthenica* exhibits extensive genetic diversity, and its genome contains numerous positive selection signals associated with stress resistance. It can tolerate multiple abiotic and biotic stresses through morphoplasticity, physiological metabolic regulation, and transcriptional regulation. Furthermore, its symbiosis with microorganisms such as rhizobia significantly enhances its stress tolerance. *M. ruthenica* demonstrates outstanding application potential in degraded grassland restoration and high-quality forage production. Future research should focus on mining stress-resistant genes, optimizing molecular breeding techniques, and integrating artificial intelligence into breeding practices. That will facilitate its transformation from a regional endemic resource to a commercially viable functional species, thereby providing robust support for ecological security and the sustainable development of grassland-based livestock husbandry in cold and arid regions.

## 1. Introduction to *M. ruthenica*

**Figure 1:**
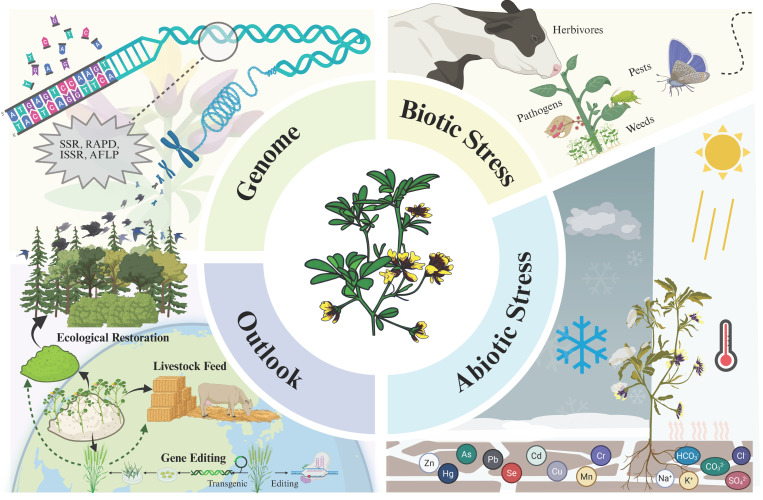
Overview of the article structure, created in BioRender. Tao, T. (2026) https://BioRender.com/hp22es1.

*Medicago ruthenica* L. (*M. ruthenica*) is a leguminous forage grass with remarkable stress tolerance, exhibiting significant adaptability under multiple adverse conditions such as drought, saline-alkali soil, and low temperatures. It is rich in phenolic compounds and possesses both antioxidant and antibacterial properties [1]. Mature plants can safely endure winters as cold as −45 °C, and seeds tolerate NaCl concentrations up to 250 mmol/L during germination under stress [2]. With the escalating challenges of global climate change and land degradation, the stress resistance mechanisms of *M. ruthenica* have garnered extensive attention, positioning it as a “strategic gene bank” for breeding stress-tolerant alfalfa. In recent years, a series of groundbreaking achievements have been made at the molecular level and other levels of research on *M. ruthenica*. This paper comprehensively reviews the latest research on *M. ruthenica*, summarizing its genetic diversity and genome, stress tolerance, utilization potential, and development prospects (Figure 1). It also compiles previously uncollated advances in genome sequencing, biostress responses, and related studies on stress-tolerant microbes. This review aims to provide a theoretical foundation for *M. ruthenica* field production and management, new variety breeding, and utilization, while offering theoretical references for ecological restoration and carbon sink agriculture in cold and arid regions.

*M. ruthenica* L. is a perennial (rarely annual) herbaceous plant belonging to the *Medicago* genus within the Fabaceae family. Formally documented and named by Linnaeus in 1753, it is now widely distributed globally, primarily concentrated across Eurasia, Africa, and South America, at elevations of 2000–4250 m. It mainly inhabits valley meadows, river gravel bars, forest-edge shrublands, and mountain slope grasslands, where it forms distinct ecotypes adapted to different habitats. Classified by growth habit, growth period, and habitat differences, *M. ruthenica* can be divided into multiple ecotypes, including the erect type (typical grasslands of central and eastern regions), salt marsh type (saline soils of Ningxia), early-maturing type (western Jilin), and Qinghai creeping type (alpine meadows at 3800 m elevation). Each inflorescence bears 4–15 flowers with yellow corollas and purplish-brown abaxial surfaces. The keel petal displays deep red stripes, and its pollen surface exhibits coarser reticulate patterns than those of alfalfa (Medicago sativa), serving as a basis for interspecific identification [3]. It exhibits a predominantly cross-pollinated hybridization system [4], with pods readily dehiscing upon maturity, each containing 2–6 seeds. Seeds are brownish-red, elliptic-ovate, and exhibit high hardseededness. The root system is a taproot–fibrous root hybrid type, with pale pink nodules present at 10–100 cm.

## 2. Genetic Diversity and Genome

### 2.1. Genetic Diversity of M. ruthenica

Research on the genetic diversity of *M. ruthenica* commenced in 2008. Using isozymes, 18 populations from the Mongolian Plateau were classified into eastern and western groups [5], which establishes a baseline for *M. ruthenica* diversity. Subsequent studies progressively deepened through phenotypic, cytological, and molecular biological analyses. Comparative evaluations of traits, including pods per plant, plant height, hay yield, seed shape, and hardseededness [6], revealed abundant wild-type variation. These phenotypes showed significant correlations with annual mean temperature, precipitation, and ecological zones [7]. *M. ruthenica*’s high genetic diversity complicates classification but endows it with rich phenotypic traits, enabling the development of superior varieties through hybridization. Studies indicate that while the F1 hybrid of erect × wild-type *M. ruthenica* exhibits a significantly higher leaf-to-stem ratio than parents, its overall heterosis remains modest. However, F2 segregation is pronounced, with ZH20 emerging as a promising new high-yielding, high-quality germplasm [8]. Genome-ecology integration analysis reveals gene flow between *M. ruthenica* and its sister species, *Medicago archiducis*-*nicolai Sirj*. The genome carries extensive signals of positive selection for drought, heat, and salt tolerance [9], making it a natural gene pool for stress-resistant breeding in cultivated alfalfa.

In-depth analysis of *M. ruthenica*’s genetic diversity using molecular marker technology indicates high genomic stability during seed storage [10,11], while the genetic base of cultivated varieties is relatively narrow [12]. Populations at plateau margins exhibit significant genetic drift effects [13], such as the northeastern Qinghai–Tibet Plateau population forming an independent branch with a low effective population size, necessitating priority conservation in its native habitat. Among molecular marker technologies, the Amplified Fragment Length Polymorphism (AFLP) system for *M. ruthenica* shows high consistency with Simple Sequence Repeat (SSR) data [10]. However, AFLP exhibits higher polymorphism efficiency while SSR demonstrates stronger geographic directionality [14], making them complementary. Based on existing reports, the genetic diversity parameters of *M. ruthenica* revealed by different molecular marker technologies are summarized to comprehensively characterize the species’ overall genetic level, as shown in Table 1.

### 2.2. Genome Sequencing of M. ruthenica

Sequencing efforts for *M. ruthenica* commenced in 2019, yielding a chromosome-based genome 904.1 Mb in size and identifying hundreds of candidate genes associated with drought tolerance [26]. In 2020, chromosome sequencing of wild-type *M. ruthenica* from Xinglong Mountain, Yuzhong County, and 37 wild populations was conducted. Analysis of single-nucleotide polymorphisms yielded a dataset of 3.939 million SNPs, confirming two racial lineages whose isolation began 55,000 years ago (during the Last Glacial Period) [27]. In 2021, the chloroplast genomes of wild accession from Xunhua County and cultivated accession from Shanxi Agricultural University were sequenced, yielding full chloroplast genome sizes of 126,939 bp and 124,254 bp, respectively [28,29], followed by a comprehensive analysis of the chloroplast genomic characteristics of *M. ruthenica* [30]. In 2023, comparison of multiple *M. ruthenica* nuclear genomic DNA barcodes revealed that the single-copy gene GA3ox1 achieved 100% identification accuracy when distinguishing *M. ruthenica* from its cryptic relative *Medicago archiducis*-*nicolai* [31].

**Figure 2:**
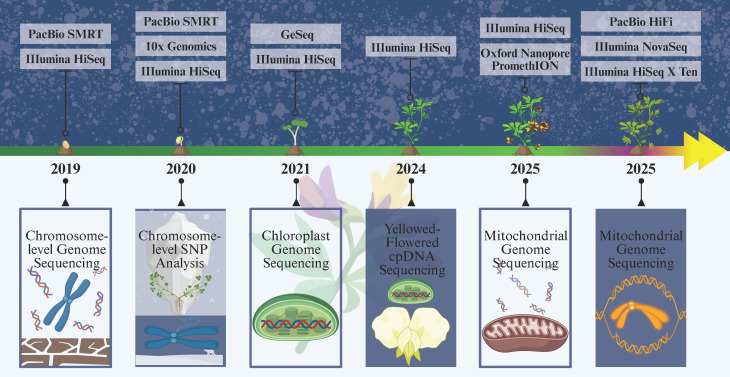
Workflow of *Medicago ruthenica* genome sequencing, created in BioRender. Tao, T. (2026) https://BioRender.com/pftnl2s.

In 2024, the chloroplast genome sequence of yellow-flowered *M. ruthenica* was determined. Its chloroplast gene preference analysis results aligned with those of common *M. ruthenica*, both showing a bias toward codons ending in A/U [32]. That same year, de novo assembly and analysis of complete chloroplast genomes from 16 cultivated germplasm accessions and 45 wild germplasm accessions of *M. ruthenica* not only established a high-quality pan-chloroplast genome but also identified highly variable regions (e.g., accD and clpP) for variety identification and phylogenetic studies [33]. In 2025, the first mitochondrial genome of *M. ruthenica* was sequenced and assembled as a 354,988 bp circular molecule rich in scattered repeats and SSRs [34], exhibiting pronounced characteristics of rearrangement and expansion. Concurrently, a 915 Mb chromosome-level genome (contig N50 = 26.26 Mb) was successfully constructed and assembled to “gold standard” quality, then resequencing, combined with multi-tissue transcriptomes and temporal expression data, revealed structural variation as a core driver of adaptive divergence between *M. ruthenica* and its sister species, *Medicago archiducis*-*nicolai Sirj*. [35]. The timeline of *M. ruthenica* genome sequencing is shown in Figure 2.

These advances collectively provide comprehensive genomic resources spanning nuclear, chloroplast, and mitochondrial genomes, enabling both evolutionary insights and practical applications in germplasm identification. The rapid progression from single-reference assemblies to pan-genome and multi-organellar datasets reflects the growing research interest in this species, while the integration of population genomics with glacial history offers a valuable perspective on Quaternary climate effects on alpine legume distribution. Nevertheless, most candidate genes identified through sequencing await functional validation.

## 3. Biological Stress Research on *M. ruthenica*

The geographic distribution, population dynamics, and ecological functions of *M. ruthenica* are constrained by complex interactions among various biological stresses, including pathogenic microorganisms (such as fungi, bacteria, and viruses), herbivorous animals and insects, and competing plants. Its susceptibility to pests and diseases primarily includes common rusts, brown spot, root rot, downy mildew, as well as aphids, thrips, weevils, stink bugs, and alfalfa leaf beetles [36]. Existing studies have successively investigated the resistance of *M. ruthenica* to downy mildew, powdery mildew, black spot, leaf spot, and rust, as well as some disease resistance mechanisms. Powdery mildew resistance can be considered one of the quality traits of *M. ruthenica*. Through morphological observation and ITS/LSU sequence analysis, the powdery mildew pathogen infecting *M. ruthenica* was identified as *Erysiphe pisi* [37]. Furthermore, significant genetic variation in resistance was observed among *M. ruthenica* germplasm, and resistance was negatively correlated with seed traits (e.g., size and plumpness) [38]. Analysis of disease incidence and severity index revealed that *M. ruthenica* exhibits higher resistance to Alternaria alternata, the causative agent of black spot disease, compared to alfalfa and *Melilotus officinalis* [39]. Furthermore, it demonstrated high resistance when inoculated with leaf spot pathogens *Diplocarpon pomorum*, *Nectria cylindrispora*, and *Parastagonospora nodorum* [40], indicating broad-spectrum quantitative resistance.

Its disease resistance mechanisms primarily involve secondary metabolite secretion and gene–pathogen interactions. The secondary metabolites responsible for antimicrobial activity are mainly flavonoids and phenolic compounds [41], exerting antibacterial effects through antimicrobial, antioxidant, and anti-inflammatory bioactivities, as well as disrupting pathogen cell membrane structures and inhibiting enzyme activity. Simultaneously, *M. ruthenica* enhances its own resistance or suppresses pathogen growth by upregulating specific genes. For example, pathogenesis-related (PR) protein genes and NOD-like receptor (NLR) genes can activate immune responses through expression [42] to resist pathogen invasion, providing targets for identifying key disease resistance genes and molecular breeding. While these studies have established foundational knowledge of *M. ruthenica* disease resistance, most remain at the phenotypic or correlational level. Functional validation of candidate resistance genes is limited, and the molecular pathways linking secondary metabolites to pathogen suppression await systematic characterization. Moreover, resistance breeding programs have yet to translate these findings into cultivar development.

Comprehensive microscopic feeding analyses of four herbivores on the Gannan grasslands revealed that the primary consumption pressure on the aboveground biomass of *M. ruthenica* stems from grazing livestock—specifically yak and Tibetan sheep [43]. Beyond grazing, livestock trampling significantly inhibits *M. ruthenica* growth, suppressing its aboveground biomass, root development, and photosynthesis [44,45,46,47], and crucially, mixed grazing further exacerbates these effects compared to single-species grazing [48].

Moreover, another covert and persistent biotic stress severely inhibits *M. ruthenica*’s population establishment and ecological niche breadth: biological competition. Seed yield losses due to weed competition during the seedling stage can reach up to 80%. Comparing the adaptability of *M. ruthenica* to invasion and colonization by the invasive species *Stellera chamaejasme* and *Solanum rostratum*, it was concluded that *M. ruthenica* is a dominant species in *S. chamaejasme*-invaded communities, maintaining high cover even as its invasion intensifies [49], indicating a tolerance threshold of *M. ruthenica* against its allelopathy and resource competition. Conversely, *S. rostratum* significantly inhibits *M. ruthenica* growth, confirmed as a sensitive species through *S. rostratum* aqueous extract allelopathy tests [50], suggesting *S. rostratum* may suppress *M. ruthenica*’s regeneration and establishment through chemical interference during community succession. Current research indicates that a two-year field experiment conducted in Inner Mongolia revealed the optimal weed control regimen for *M. ruthenica* is 1530 mL ha^−1^ of imazethapyr or 120 mL ha^−1^ of azoxystrobin pre-emergence, followed by post-emergence application of imazethapyr +fluazifop-p-butyl (1800 + 600 mL ha^−1^) or 2,4-DB+fluazifop-p-butyl (2250 + 600 mL ha^−1^) [51]. This regimen achieves effective weed control without significantly impairing *M. ruthenica* growth or adversely affecting soil microorganisms.

Despite progress in identifying biotic stress factors, integrated management strategies remain underdeveloped. Most studies address single stressors in isolation, whereas field conditions involve simultaneous pathogen, herbivore, and competitor pressures. Additionally, the long-term ecological consequences of chemical weed control on *M. ruthenica* population dynamics and community stability require further assessment.

## 4. Abiotic Stress Research on *M. ruthenica*

**Figure 3:**
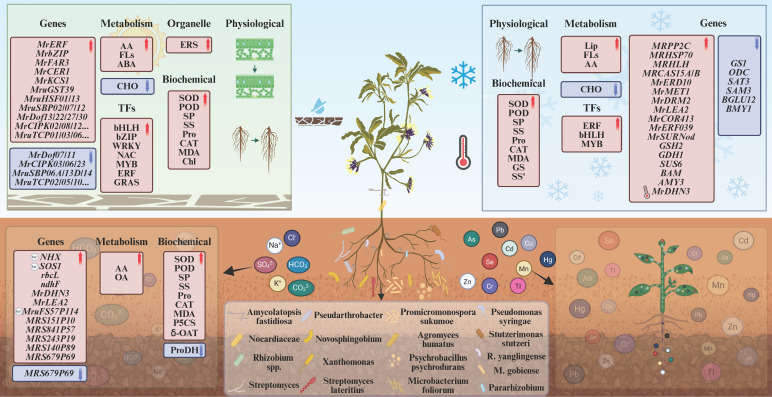
Schematic representation of the multi-dimensional stress response mechanisms in *Medicago ruthenica*. The diagram illustrates the physiological, biochemical, and molecular adaptations of *M. ruthenica* under four major abiotic stresses: () **Drought Stress** (top left), characterized by the regulation of stomatal movement, osmotic adjustment via proline and soluble sugars, and the activation of DREB/ERF transcription factors; () **Temperature Stress** (top right), involving membrane lipid remodeling, antioxidant enzyme (SOD, POD) activation, and the expression of cold-responsive genes (e.g., *MrCOR*, *MrCBF*); () **Salt Stress** (bottom left), highlighting ion homeostasis maintenance (Na^+^/K^+^ balance) and the synthesis of osmoprotectants; and () **Heavy Metal Stress** (bottom right), depicting chelation, sequestration, and detoxification processes. The central panel emphasizes the role of beneficial root-associated microorganisms (e.g., *Rhizobium*, arbuscular mycorrhizal fungi (AMF)) in enhancing systemic stress tolerance through nitrogen fixation, nutrient solubilization, and the modulation of phytohormone signaling. Created in BioRender. Tao, T. (2026) https://BioRender.com/6ekbej8.

As a legume with multiple abiotic stress tolerances, *M. ruthenica* serves as a promoted species for cultivation and restoration in saline-alkali and arid lands. Research on its stress resistance mechanisms has primarily focused on morphological anatomy, physiological biochemistry, metabolism, and cellular and molecular levels. Its seeds maintain high germination rates across a temperature range of 10–40 °C and tolerate salinity-alkalinity up to 200 mM NaCl or 25 mM Na_2_CO_3_ [2]; seeds that fail to germinate under stress can rapidly resume germination once stress is relieved. During the seedling stage, *M. ruthenica* achieves ecological adaptation through morphoplasticity: high-fertility, high-moisture wetlands promote large-leaf, high-yielding plant types, while sandy, low-fertility, arid conditions select for stress-resistant, tall-branching forms [52]. Stress resistance is further enhanced by increasing alkane chain length or synthesizing specialized wax components [53]. Based on 128 resequenced samples, multi-tissue transcriptomes, and cold/heat stress time-series expression data, structural variations (SVs) were identified as a core driver of *M. ruthenica* adaptive differentiation [35]. Comparative analysis of *M. ruthenica* unigene expression profiles under cold, freezing, osmotic, salt, and abscisic acid (ABA) stress identified 2721 stress-responsive genes. Their functions primarily focused on reactive oxygen species (ROS) scavenging, plant hormone signaling transduction, and regulatory networks involving transcription factors such as *APETALA2/Ethylene Response Factor (AP2/ERF)*, *Myeloblastosis (MYB)*, *WRKY*, and *NAM/ATAF1/2/CUC2 (NAC)* [54]. Subsequent transgenic studies demonstrated that the *MrERF/MrbZIP* genes from *M. ruthenica* enhance drought and salt tolerance in transgenic tobacco, while MrSURNod significantly improves cold tolerance. These three genes synergistically enhance *M. ruthenica*’s adaptation to multiple abiotic stresses by promoting root development, strengthening osmotic regulation, and bolstering antioxidant systems [55]. Synthesizing progress in *M. ruthenica* abiotic stress tolerance research (Figure 3) illustrates its potential as a donor of stress-resistance genes, offering valuable genetic resources and theoretical guidance for rapidly developing new salt- and cold-tolerant varieties by introducing these superior stress-resistant genes into elite related species through molecular breeding.

### 4.1. Drought Tolerance

As a mesophytic plant, *M. ruthenica* possesses a series of efficient drought response mechanisms. Morphological and structural adaptations—specifically the thickening of palisadex tissue, thinning of spongy tissue, leaf thickening, improvement of tissue compactness, and enhancement of vein protrusion—are recognized as key indicators to assess plant drought resistance [2]. As a taproot-type C3 plant, *M. ruthenica* enhances drought resistance by increasing primary root length to improve water uptake in drought and coarsening soils [56,57], exhibiting particularly higher drought tolerance in sandy soils [58]. Through high-throughput sequencing and transient co-expression experiments, it was revealed that under drought-mimicking osmotic stress, *M. ruthenica* maintains root homeostasis and recovery capacity by negatively regulating TCP4 expression via miR319 [59]. In summary, under drought stress, *M. ruthenica* prioritizes allocating organic matter and nutrients to the roots to promote root growth and absorb more water, thereby enhancing its adaptability. Concurrently, prolonged drought stress increases malondialdehyde (MDA) content in *M. ruthenica*, while superoxide dismutase (SOD), peroxidase (POD), chlorophyll, soluble sugar (SS), soluble protein (SP), and proline (Pro) initially rise and then decline, recovering to control levels upon rehydration [60], demonstrating strong rehydration sensitivity and potential recovery capacity. These findings establish the physiological basis of drought adaptation in *M. ruthenica*, yet most observations remain descriptive, lacking quantitative links between specific traits and field performance under variable drought intensities.

Researchers systematically revealed multiple regulatory pathways in the molecular response of *M. ruthenica* to drought stress using metabolomics and transcriptomics, primarily encompassing four hierarchical levels: “metabolism-MAPK-miRNA-transcription factor-functional genes.” Metabolite differential analysis revealed that *M. ruthenica* primarily enhanced drought tolerance by increasing carbohydrate (CHO) and amino acid (AA) metabolism on day 9 of drought stress. By day 12, adaptation to drought conditions was achieved mainly through regulating its osmotic signaling system and photosynthesis [61]. ABA and auxin signaling pathways serve as key regulators in *M. ruthenica*’s drought response. Concurrently, flavonoid (FL) synthesis and circadian rhythm regulation accelerate its recovery process. Multi-omics analysis reveals that under drought stress, *M. ruthenica* utilizes a multi-layered “hormone-MAPK-miRNA” network to synergistically regulate transcriptional and post-transcriptional processes, enhancing drought tolerance. For instance, the mtr-miR156b-5p pathway is significantly upregulated during drought stress (and downregulated after rehydration), forming a post-transcriptional regulatory switch [62]. Furthermore, after just two cycles of drought rehydration, *M. ruthenica* establishes low-methylation stress memory, enhancing expression of ABA and proline synthesis genes [63] to accelerate responses to subsequent drought stress. While these multi-omics studies have constructed comprehensive regulatory frameworks, most candidate genes await functional validation, and the translational gap between laboratory findings and cultivar development remains substantial.

Analysis of the *M. ruthenica* transcriptome under drought stress systematically identified multiple transcription factor families, including Ethylene Response Factor (*ERF*), basic Helix-Loop-Helix (*bHLH*), basic Leucine Zipper (*bZIP*), *NAC*, *MYB*, *WRKY*, and *GRAS*. Members such as *MrGRAS11/24/29* [64] are significantly induced by drought, providing key candidate genes and molecular markers for subsequent functional validation and stress-tolerant forage breeding. Using transcriptomic and weighted gene co-expression network analysis (WGCNA) techniques to identify drought-responsive genes in *M. ruthenica*, 18 core genes enriched in ABA/JA signaling, endoplasmic reticulum stress (ERS), protein homeostasis, and antioxidant metabolism were identified [65]. Specifically, genes such as *MrFAR3-1*, *MrCER1*, *MrFAR3-2*, and *MrKCS1* were found to promote cuticle wax synthesis, thereby enhancing drought resistance in *M. ruthenica* [66]. Additionally, transcriptome analysis revealed the upregulation of drought-responsive genes, namely *MrERF*, *MrbZIP* [62], *MruSBP02/07/12* [67], *MruTCP01/03/06/08/09/17/18* [68], *MrDof13/22/27/30* [69], *MrCIPK02/08/12/16/22* [70], *MruHSF01/13* [71], and 10 *MruGST* genes [72], alongside the downregulation of *MruSBP06A/13D/14* [67], *MruTCP02/05/10/16/19* [68], *MrDof07/11* [69], *MrCIPK03/06/23* [70], and 4 *MruGST* genes [72]. Among these, *MrDof11/13/22/27/30* and *MrCIPK02* emerged as core candidate genes regulating drought tolerance in *M. ruthenica*. Agrobacterium-mediated overexpression of *MruGSTU39* in both *M. ruthenica* and alfalfa resulted in higher survival rates under drought stress compared to the controls in both species [72]. Despite the identification of numerous candidate genes, only a handful have undergone functional validation.

### 4.2. Salt–Alkali Tolerance

*M. ruthenica* has demonstrated highly efficient adaptive mechanisms in studies of salt–alkali stress tolerance. Current research on its salt–alkali resistance primarily focuses on phenotypic, physiological, metabolic, and genetic levels. The growth parameters (plant height, leaf area, and stem diameter) of *M. ruthenica* exhibit a continuous decline with increasing salt concentration, indicating significant inhibition. Concurrently, the root-to-shoot ratio increases markedly [73], suggesting that *M. ruthenica* may respond to salt stress by elongating roots. The decrease in K^+^ concentration in roots, followed by a reduction in stem and leaf K^+^ content, indicates that *M. ruthenica* may mitigate salt damage by redistributing K^+^ within the plant. Existing physiological studies reveal that in *M. ruthenica*, MDA, SOD, Pro, leaf relative electrical conductivity, Δ^1^-pyrroline-5-carboxylate synthetase (P5CS) activity, and ornithine-δ-aminotransferase (δOAT) activity all exhibit an upward trend with increasing salt concentration or prolonged stress duration, while chlorophyll, SP, SS, leaf relative water content (RWC), and proline dehydrogenase (ProDH) activity show decreasing trends; additionally, catalase (CAT) activity first increases then decreases [73,74,75]. This indicates that under saline–alkali stress, *M. ruthenica* drives its antioxidant system by accumulating MDA, enhances membrane permeability to accelerate Na^+^ efflux and osmotic signaling, synergistically upregulates P5CS/δOAT activity, and downregulates ProDH activity. This achieves rapid proline accumulation and a dynamic equilibrium between chlorophyll and RWC, thereby enhancing its salt tolerance. Analysis of metabolic profiles under saline–alkali stress revealed that under 0.3% mixed salts, *M. ruthenica* primarily relies on accumulating AAs and their derivatives alongside organic acids (OA) to remodel osmotic and antioxidant systems [76], thereby sustaining normal seed germination.

*M. ruthenica*’s molecular-level research has primarily focused on identifying and validating salt-tolerant genes. For instance, Hou et al. [77] identified six salt-tolerant candidate genes in *M. ruthenica* through transcriptomic analysis: *MR S151P10*, *MR S841P57*, *MR 51P182* (*SPECHLESS*), *MR S243P19*, *MR S140P89*, and *MR S679P69*. Meanwhile, *MruFS57P114* was demonstrated to enhance salt tolerance by inhibiting *High-affinity K^+^ Transporter 1 (HKT1)*, reducing Na^+^ influx, and mitigating oxidative damage [78]. Studies showed that compared to controls, *Escherichia coli* transformed with *MrDHN3* and *MrLEA2* genes exhibited significantly higher surviving colony counts in LB medium containing 0.5 mol/L NaCl and 0.5 mol/L KCl [79,80], indicating that overexpression of *MrDHN3* and *MrLEA2* enhances E. coli salt tolerance. Concurrently, positive selection has been observed on salt–alkali stress-related genes (e.g., *ribulose-1,5-bisphosphate carboxylase/oxygenase large subunit (rbcL)*, *NADH dehydrogenase subunit F (ndhF)* [33]) within the chloroplast genome of *M. ruthenica*. This indicates that plastid-encoded gene variants can enhance photosynthetic stability under combined salt–alkali stress, offering novel insights for designing genetically improved, high-quality salt-tolerant cultivars.

In summary, research on *M. ruthenica* salt–alkali tolerance has progressed from physiological description to gene discovery, yet significant gaps persist between laboratory findings and field application. Most studies employ simplified stress regimes, and functional validations rely heavily on heterologous systems. Future efforts should prioritize stable genetic transformation in *M. ruthenica*, field-based evaluation of candidate genes, and integration of physiological markers with molecular breeding strategies.

### 4.3. Temperature Stress Tolerance

Temperature stress is categorized into two main types: low-temperature stress and high-temperature stress. Both disrupt plant homeostasis through distinct mechanisms, constraining plant distribution ranges and productivity. Among these, low-temperature stress is the primary factor limiting plant geographic distribution and seasonal adaptability. During the early growth stage of *M. ruthenica*, low-temperature stress inhibits seed germination. Comparing the germination responses of three *M. ruthenica* cultivars at 0 °C, 5 °C, 15 °C, 25 °C, and 30 °C, a comprehensive evaluation of cold tolerance revealed the following hierarchy: wild-type > early-maturing > erect-type [81]. During the seedling stage, low-temperature stress significantly inhibits *M. ruthenica*’s height growth while promoting root elongation [82]. Analysis of physiological metabolism revealed increased levels of SS, SP, D-trehalose, D-sucrose, maltose, lipid (lip), flavonoid (FL), AAs and their derivatives, and glutamine synthetase (GS) activity. Conversely, starch and MDA content decreased. POD activity first increased, then decreased, while sucrose synthase (SUS) activity first decreased, then increased [83]. Additionally, the carbon and nitrogen isotope abundances and total carbon content of *M. ruthenica* were higher than controls, indicating that it enhances cold tolerance through dual hydrolysis of starch and protein and enhances antioxidant capacity and membrane stability, supplemented by lipid/flavonoid-mediated membrane restructuring and regulation of carbon and nitrogen metabolism.

Research on the molecular response mechanisms of *M. ruthenica* cold tolerance began in 2013. Under low-temperature stress, *M. ruthenica* upregulates the expression levels of genes including *MRPP2C*, *MRCAS15A/B*, *MRHSP70*, *MRHLH*, *Glutathione Synthetase 2* (*GSH2*), *Glutamate Dehydrogenase 1* (*GDH1*), *Sucrose Synthase 6* (*SUS6*), *β-Amylase 3* (*BAM3*), *α-Amylase 3* (*AMY3*), *MrCOR413*, *MrP5CS* and *MrERD10*, along with transcription factors from families such as *ERF*, *bHLH* and *MYB*. Simultaneously, it downregulates the expression of genes including *β-Amylase 1* (*BMY1*), *Glutamine Synthetase 1* (*GS1*), *Ornithine Decarboxylase* (*ODC*), *S-Adenosylmethionine Synthetase 3* (*SAM3*), *Serine Acetyltransferase 3* (*SAT3*) and *β-Glucosidase 12* (*BGLU12*) to exert cold tolerance [83,84,85]. Notably, *MRHLH* and *MRCAS15A/B* showed significant positive correlations with SOD enzyme activity [84]. The *MrERF039* gene functions as a sugar molecular switch, influencing the expression of sugar transporters and sugar metabolism-related genes through transcriptional regulation. It also directly regulates the expression of β-amylase genes, UDP-glucosyltransferase genes, and C2H2-type zinc finger protein genes [85], synergistically enhancing the cold tolerance of *M. ruthenica* under low temperatures, preliminarily revealing the complex network of intracellular signaling regulation during cold resistance in erect *M. ruthenica*. The relative expression levels of *MrMET1* and *MrDRM2* genes in *M. ruthenica* accumulated to 3.7- and 4-fold, respectively, compared to controls after 12 h of 4 °C cold stress. Following −20 °C freezing treatment, the colony count of E. coli expressing the *MrLEA2* gene was significantly higher than the control group, collectively indicating that these cold-inducible genes play pivotal roles in the enhanced freezing tolerance of *M. ruthenica*. [80,86,87].

Currently, research on *M. ruthenica* responses to high-temperature stress is still in its infancy, with substantial gaps at physiological and omics levels. Some scholars have predicted, using MaxEnt models and ecological theory, that the suitable habitat range of *M. ruthenica* is likely to shrink significantly under future climate warming [88]. However, this inference may overlook its adaptive evolutionary potential and phenotypic plasticity under high-temperature conditions. Research indicates that Escherichia coli strains heteroexpressing the *M. ruthenica* SK2-type dehydrogenase gene *MrDHN3* exhibited significantly higher survival rates than controls after 30 min of 55 °C heat treatment [79]. This demonstrates that heteroexpression of *MrDHN3* substantially enhances E. coli resistance to high-temperature stress, suggesting the gene may participate in cellular protection mechanisms under heat stress.

Collectively, *M. ruthenica* cold tolerance research has progressed from physiological description to multi-layered molecular networks, yet functional validation and mechanistic integration remain incomplete. Conversely, high-temperature responses are virtually unexplored beyond single-gene heterologous assays—a critical imbalance given climate change projections.

### 4.4. Heavy Metal Tolerance

Few studies have reported on the heavy metal tolerance of *M. ruthenica*. Existing research primarily focuses on the effects of heavy metal stress on growth indicators, its tolerance to heavy metals, and the influence of fertilizer application on its efficacy in heavy metal phytoremediation. The germination rate, shoot length, and root length of *M. ruthenica* all decrease with increasing concentrations of heavy metals such as copper (Cu^2+^), cadmium (Cd^2+^), and lead (Pb^2+^) [89]. However, it also exhibits certain tolerance and accumulation potential. Subsequent studies revealed that *M. ruthenica* exhibits good adsorption capacity for arsenic (As), mercury (Hg), cadmium (Cd), lead (Pb), selenium (Se), copper (Cu), zinc (Zn), and manganese (Mn). It also demonstrated survival in chromium (Cr) and nickel (Ni)-contaminated areas [90], exhibiting good absorption, accumulation, and transport of heavy metals. However, its low biomass and short stature resulted in reduced heavy metal remediation capacity per unit area. Comparing the effects of different fertilizers on the remediation of heavy metal (Cd, As, Ti) contamination in sandy soils within mining areas using *M. ruthenica*, the “nano-humus + arbuscular mycorrhizal fungi (AMF)” combination not only significantly reduced soil Cd and As levels in the soil, but also significantly increased the biomass, cation exchange capacity, organic carbon, and available nitrogen, phosphorus, and potassium content of *M. ruthenica* in the following year [91]. This promotes rapid vegetation restoration, heavy metal immobilization, and soil fertility recovery, demonstrating significant application value. It is evident that *M. ruthenica* possesses the core capability of “high transfer-high metal tolerance,” with its remediation limitation being biomass size rather than extraction efficiency. Subsequent strategies, such as genetically modifying tall cultivars and applying exogenous nano-humic substances like AMF, can increase metal extraction per unit area, positioning *M.* *ruthenica* as a “functional pioneer plant” for addressing complex pollution in cold, high-altitude mining regions.

Despite these promising agronomic findings, fundamental research on the heavy metal tolerance of *M. ruthenica* remains scarce. Existing studies are largely confined to phenotypic descriptions of growth responses and preliminary assessments of accumulation capacity [89,90]. Notably, no studies have yet explored the underlying molecular mechanisms—such as specific metal transporter identification, detoxification pathway characterization, or transcriptional regulatory networks—that govern its multi-metal tolerance and hyperaccumulation traits. This knowledge gap severely limits the predictive capacity for genotype–environment matching and hinders targeted genetic improvement for phytoremediation applications. Therefore, while *M. ruthenica* is positioned as a “functional pioneer plant” with a core capability of “high transfer-high metal tolerance,” future strategies must move beyond agronomic amendments (e.g., exogenous nano-humic substances + AMF) to include the genetic dissection of these tolerance traits, potentially enabling the breeding of high-biomass cultivars suitable for complex pollution in cold, high-altitude mining regions.

### 4.5. Rhizobia-Mediated Stress Tolerance

Leguminous forage crops exhibit remarkable adaptability in poor, arid, and other adverse soils. This resilience stems not only from their inherent stress tolerance mechanisms but also from their symbiotic relationship with rhizobia. Rhizobia can convert atmospheric nitrogen into ammonium nitrogen via nitrogenase and secrete active substances such as extracellular polysaccharides (EPS) [92], thereby enhancing the stress tolerance of *M. ruthenica*. For instance, the rhizobium GBXD30 significantly improves *M. ruthenica*’s ability to adapt to low-temperature stress by increasing antioxidant enzyme activity and nodule nitrogen fixation capacity [93]. Under prolonged mowing disturbance, *M. ruthenica* also maintains nutritional balance in nitrogen-depleted grassland environments by strengthening its symbiotic nitrogen-fixing relationship with rhizobia [94]. Statistical analysis indicates that the co-occurrence link density within the rhizosphere bacterial network of *M. ruthenica* ranks among the highest, suggesting it enhances ecological adaptability to stress by constructing a rhizosphere microbial network with high interaction intensity. Different genotypic *M. ruthenica* varieties significantly influence stress resistance by selectively recruiting rhizosphere microbes. For instance, inoculation with microbes isolated from highly drought-tolerant *M. ruthenica* strains like Mengnong 2 effectively enhances *M. ruthenica*’s drought tolerance [95,96]. Furthermore, the resistance of symbiotic bacteria to environmental stress is a key factor affecting their practical application and adoption in agricultural production. Twenty-six cold-tolerant plant growth-promoting bacteria (PGPB) strains were screened from the rhizosphere/endosphere of *M. ruthenica*. Among them, strains such as MYnA5, MYpAn1, MYmA3, and MYnD1 have been confirmed to significantly promote the plant height of *Elymus nutans* Griseb [97,98], serving as candidate strains to enrich the germplasm resource bank of agricultural beneficial microorganisms in cold regions. The bacterial strains capable of enhancing the stress resistance of *M. ruthenica* are summarized in Table 2.

While these studies demonstrate the functional potential of rhizobia and PGPB in enhancing *M. ruthenica* stress tolerance, mechanistic understanding of these microbe–plant interactions remains superficial. Most evidence derives from correlation-based inoculation assays [93,94,95,96], lacking molecular characterization of signaling pathways, metabolic exchanges, or strain-specific colonization mechanisms. Moreover, the transition from laboratory-screened strains [97,98] to field-applicable inoculants is hindered by uncharacterized ecological compatibility and competitive fitness in native soil microbiomes.

Nevertheless, these microbial resources hold significant promise for developing commercial biofertilizers tailored to cold and arid regions, offering sustainable alternatives to chemical nitrogen fertilizers while improving forage establishment in degraded grasslands. Future efforts should prioritize formulation optimization and field-scale validation to realize their agricultural potential.

## 5. Development and Utilization

*M. ruthenica* is a wild relative of cultivated alfalfa that provides a reservoir of stress-resistant alleles, stabilizes grassland ecosystems in cold and arid regions, and yields forage rich in crude protein. It offers both germplasm and reference protocols for breeding stress-resistant alfalfa, restoring degraded grasslands, and updating the forage-based livestock systems.

### 5.1. Genetic Resource Value

The first interspecific hybrid of *M. ruthenica* was produced in 1987. Following multi-stage screening for spring-freeze tolerance and subsequent radiation mutagenesis, the high-yield cold-hardy cultivars Longmu 801 and 803 were released [101], establishing the species as a breeding crop. A core set of 28 accessions, capturing the full phenotypic spectrum of 113 collections, is now available for targeted breeding [102]. Elucidating the chemotaxonomy and anthocyanin pathway of yellow-flowered *M. ruthenica* delivers both rationale and targets for engineering flower color and developing novel color variants [103]. A short-cycle, tissue culture-independent Agrobacterium rhizogene-mediated hairy-root transformation protocol of *M. ruthenica* has been established [104]. This system can be directly applied to salt and drought stress assays and has been successfully adapted to CRISPR/Cas9 vectors in alfalfa, offering a ready platform for rapid functional gene validation and molecular breeding of leguminous forages. A complete pipeline involving germplasm mining, mechanism analysis, technical validation, and directed breeding has been assembled for *M.* *ruthenica*, providing a transferable template for genetic improvement of allied species.

### 5.2. Degraded Grassland Restoration

Restoring degraded grasslands is a core component of ecological management in cold and arid regions. With efficient N-fixation, low input demand, and high stress tolerance, *M. ruthenica* is a prime candidate for near-natural recovery. Its deep root system effectively stabilizes surface soil and reduces erosion. Meanwhile, via symbiosis with rhizobia, it converts atmospheric nitrogen into plant-available forms to enhance soil fertility, acting as both a soil ameliorant and a plant community stabilizer in degraded grasslands [105]. High root nitrogen concentration, low priming effect, and strong carbon input capacity give *M. ruthenica* a net gain in soil organic carbon and make it a key regulator of soil carbon sinks [106]. By enriching beneficial microorganisms, *M. ruthenica* forms positive “soil memory” that fosters subsequent plant growth and diversity, enhancing ecosystem multifunctionality [107]. For instance, overseeding degraded alpine meadows with the *M. ruthenica* cultivar ‘Longzhong No. 1’ relieves nitrogen limitation through nitrogen fixation via rhizobial symbiosis, enhances nitrogen acquisition capacity in companion grasses, and suppresses toxic weed expansion by rapidly occupying ecological niches [108].

Utilizing *M. ruthenica* for degraded grassland restoration significantly reduces artificial fertilization and irrigation costs. Its biological nitrogen fixation capacity minimizes chemical nitrogen fertilizer inputs while enhancing soil carbon sink functions, offering potential revenues through carbon trading and achieving synergistic ecological and economic benefits. Moreover, both *M. ruthenica* itself and its rhizosphere soil extracts are rich in short-chain organic acids, flavonoids, and terpenoids [109], exhibiting specific allelopathic potential. This synergistically promotes forage growth while inhibiting toxic weed proliferation, providing valuable allelopathic material for restoring degraded alpine grasslands.

### 5.3. Forage Value

With the rapid expansion of grass-product markets, specializing in forage seed production is now an urgent priority [110]. As a native leguminous forage with outstanding stress tolerance, *M. ruthenica* features tender stems and leaves, a pleasant aroma, high crude protein content (up to 15.81%), and no saponins, conferring excellent nutritive value, safety, and palatability [111]. Compared to *Leymus chinensis*, *M. ruthenica* exhibits significantly higher concentrations of crude protein, concentrated tannins, ammonia-N, and branched-chain volatile fatty acids, while exhibiting lower neutral/acid detergent fiber and methane production [112]. This allows for increased inclusion in ruminant diets to leverage its high tannin content and low methane emissions for emission reduction, efficiency gains, and high-quality forage formulation. As a shade-tolerant C3 legume, *M. ruthenica* is well-suited for intercropping and can significantly enhance hay yields in mixed grasslands through nitrogen fixation with rhizobia and secretion of secondary metabolites [113]. Large-scale cultivation of *M. ruthenica* as forage reduces feed costs while its high protein and low methane emission characteristics improve livestock production efficiency and product value-added. This simultaneously decreases greenhouse gas emissions, aligning with green breeding trends and enhancing market competitiveness and economic returns for animal husbandry.

In practical production, *M. ruthenica* faces challenges: low natural germination rates caused by high seed hardness, a low-growing, prostrate growth habit unsuitable for mechanical harvesting due to pods borne close to the ground, severe seed yield reduction from pod shattering [114], and a lack of standardized cultivation systems. *M. ruthenica* seed hardening occurs 33–36 days after peak flowering [115], with hard seed rates reaching 88.00–98.67% [116]. Pre-planting treatments to break seed hardening primarily include mechanical methods (grinding, cutting), chemical methods (H_2_SO_4_, HCl, NaOH, H_2_O_2_, KMnO_4_, KNO_3_, GA), and physical methods (electric current, temperature) [117,118,119]. Among these, grinding and cutting treatments completely break seed hardness, representing the optimal methods, followed by immersion in concentrated sulfuric acid. Genetic dissection of the erect phenotype in *M. ruthenica* revealed a single-base mutation that attenuates miR397a expression [120]. Reduced miR397a relieves repression of LAC17, a key lignin-polymerizing enzyme, promoting lignin deposition and erect stem formation. However, excessive lignin deposition is one of the drivers of *M. ruthenica* pod shattering: the lignin-enriched abscission layer (AL) stiffens the endocarp and predisposes the AL to rupture. While the other driver is the up-regulation of polygalacturonase/cellulase (PG/CE) genes, which degrade the middle lamella of AL, forming a MYB-POD-auxin network [121,122,123]. Therefore, when breeding upright varieties by enhancing lignin synthesis, the consequent effects on *M. ruthenica* pod shattering must be assessed. Varieties suitable for large-scale production await breeding, and optimal *M. ruthenica* cultivation practices (e.g., mulching regimes, fertilization schemes, and harvest timing) remain to be systematically quantified. Preliminary studies indicate that straw mulching with flat cultivation is the optimal seed production strategy for *M. ruthenica* in central Gansu and analogous arid regions [124,125]. Among nitrogen–phosphorus fertilizer interactions, the N1P2 treatment (47 kg N ha^−1^ + 120 kg P_2_O_5_ ha^−1^) yielded the most significant increases in seed production and plant growth, with the highest quality [126]. Biofertilizer assays showed that dual inoculation with *Funneliformis mosseae* and *Rhizobium* sp. CCBAU13029, or triple inoculation further adding Bacillus megaterium ACCC1001, yielded the strongest promotion of aboveground biomass in *M. ruthenica* [127]. Experimental results demonstrate that the optimal seed harvest window for *M. ruthenica* is identified as “36 days after peak flowering, combined with daily harvesting before 08:00 AM or after 6:00 PM daily” [128], whereas the optimal harvest timing for other biomass components remains undefined. Further integrated studies on *M. ruthenica*’s genetics, physiology, and agronomy for production adaptation are still warranted.

## 6. Prospects and Outlook

This review primarily synthesizes published literature without incorporating unpublished data or grey literature, which may introduce publication bias. Furthermore, heterogeneity exists across studies in experimental design, stress treatment conditions, and evaluation systems, limiting cross-study comparisons and the derivation of generalizable conclusions. Despite these constraints, research on *M. ruthenica* has advanced from phenotypic and physiological studies to genomic analysis, functional validation of genes, and molecular breeding. However, key limitations remain to be addressed: stress resistance mechanisms remain fragmented; the genetic and regulatory bases of traits such as erect growth and pod shattering resistance remain elusive; efficient transformation systems are still underdeveloped; and industrialization technologies require further integration and optimization.

To overcome these bottlenecks and propel *M. ruthenica* from a regional specialty resource to a functional crop species, the following can be carried out: expanding and phenotyping germplasm collections through high-throughput platforms and establishing standardized stress evaluation protocols; identifying stress-resistant loci and alleles via pan- and landscape genomics and large-scale GWAS leveraging natural variation panels to pinpoint major-effect QTLs for drought, cold, and low-nitrogen tolerance, followed by functional validation through CRISPR-based gene editing and transgenic complementation assays; enlarging mutant libraries and hybrid populations by mutagenesis and hybridization techniques with priority given to erect architecture and pod shattering-related mutants; screening target genes through genomic selection and integrating transcriptome-guided marker-assisted selection to accelerate elite line development; and establishing robust genetic transformation and dual gene editing platforms for *M. ruthenica*. Particularly, candidate genes identified from multi-omics analyses—including lignin biosynthesis regulators for pod shattering resistance and miRNA-mediated modules for plant architecture—should undergo systematic validation via stable transformation, dual-luciferase reporter assays, and yeast two-hybrid systems to confirm their regulatory functions and interaction networks prior to breeding deployment. These resources will enable molecular design breeding that simultaneously edits erect architecture and pod-shatter resistance, creates pyramids of multiple stress-resistance loci, reduces anti-nutritional factors (tannins, saponins, etc.), and boosts vitamin and amino acid contents, thereby achieving the synergistic improvement of stress resistance, high yield, and superior quality. Molecular discoveries should be rapidly channeled into breeding pipelines through marker-assisted backcrossing and genomic selection, establishing a “discovery-to-cultivar” continuum that bridges basic research and commercial variety release. In parallel, artificial intelligence algorithms that integrate multi-omics, phenomics, and environmental data will deliver gene function prediction, trait–environment interactions, and breeding-simulation models, shifting breeding decisions from empirical to data-driven and establishing *M. ruthenica* as a cornerstone species for ecological barrier construction in arid-cold regions, sustainable forage–livestock development, and next-generation cultivar innovation.

## Figures and Tables

**Table 1:** Genetic diversity parameters based on molecular markers.

Materials	Origins	Sampling Number	Molecular Marker	Shannon’s Polymorphism Information Index (I)	Nei’s Gene Diversity Index (H)	Diversity Among Populations (Gst)	Reference
Wild *M. ruthenica*	13 regions of Inner Mongolia, China	50 plants	SSR	0.4474	0.2891	0.5264	[15]
Four populations of *M. ruthenica*	South Suburb Experimental Farm, Hohhot, Inner Mongolia, China	148 plants	SSR	0.3639	0.2222	-	[16]
Four *M. ruthenica* lines	Sharqin Experimental Station, Institute of Grassland Research, Chinese Academy of Agricultural Sciences	80 plants	RAPD	0.4145	0.2662	-	[17]
Four *M. ruthenica* lines	Sharqin Experimental Station, Institute of Grassland Research, Chinese Academy of Agricultural Sciences	80 plants	ISSR	0.4060	0.2569	-	[12]
Wild and erect *M. ruthenica*	Sharqin Experimental Station, Institute of Grassland Research, Chinese Academy of Agricultural Sciences	44 plants	SSR	0.382	0.237	0.474	[18]
*M. ruthenica*	Inner Mongolia, Beijing, Shanxi, Hebei, Liaoning, Jilin, and Tibet, China	59 populations	ISSR	0.4870	0.3297	0.8349	[19]
*M. ruthenica*	Inner Mongolia, Beijing, Shanxi, Hebei, Liaoning, Jilin, and Tibet, China	59 populations	AFLP	0.3556	0.2138	0.4681	[19]
Eight populations of *M. ruthenica*	Sharqin Experimental Station, Institute of Grassland Research, Chinese Academy of Agricultural Sciences	8 populations	ISSR	0.3037	0.1989	-	[20]
Three ecotypes of wild *M. ruthenica*	Sharqin Experimental Station, Institute of Grassland Research, Chinese Academy of Agricultural Sciences	14 plants	ISSR	0.522	0.352	-	[21]
Three ecotypes of wild *M. ruthenica*	Sharqin Experimental Station, Institute of Grassland Research, Chinese Academy of Agricultural Sciences	14 plants	SSR	0.438	0.284	-	[21]
*M. ruthenica*	Hohhot, Inner Mongolia, China	30 plants	ISSR	0.487	0.329	-	[22]
*M. ruthenica*	Hohhot, Inner Mongolia, China	30 plants	SSR	0.372	0.231	-	[22]
*M. ruthenica*	Inner Mongolia, Beijing, Shanxi, Hebei, Liaoning, Jilin, and Tibet, China	49 populations	ISSR	0.492	0.336	-	[23]
*M. ruthenica*	Tibet, Mongolia, Chifeng, Ulanqab, Hohhot, and Baotou, China	5 plants	ISSR	0.204	0.131	-	[24]
*M. ruthenica*	Inner Mongolia, Beijing, Shanxi, Hebei, Liaoning, Jilin, and Tibet, China	49 plants	AFLP	0.4950	0.3230	-	[25]

**Table 2:** Beneficial microbiota of *Medicago ruthenica*.

Taxon	Genus Name	Strain	Separation Sites	Functions	Reference
bacteria	*Amycolatopsis*	*Amycolatopsis fastidiosa*	Root, stem, leaf, flower	putative antibiotic producer	[99]
	*Nocardia*	*Nocardiaceae*	root, stem, leaf, flower	stably colonizes the plant interior	[99]
	*Rhizobium*	*Rhizobium* spp.	rhizosphere soil	sustains nitrogen fixation and biomass accumulation under drought	[95]
	*Streptomyces*	*Streptomyces*	rhizosphere soil	produces antioxidant secondary metabolites that alleviate drought-induced oxidative stress	[95]
	*Streptomyces*	*Pseudarthrobacter*	rhizosphere soil	produces antioxidant secondary metabolites that alleviate drought-induced oxidative stress	[95]
	*Sphingomonas*	*Novosphingobium*	rhizosphere soil	modulates drought-resistance phenotypes	[95]
	*Xanthomonas*	*Xanthomonas*	rhizosphere soil	produces exopolysaccharides that enhance rhizosphere water retention and contribute to drought-adaptive strategies	[95]
	*Streptomyces*	*Streptomyces lateritius*	rhizosphere soil	solubilizes phosphate, mobilizes potassium, and tolerates drought	[96]
	*Promicromonospora*	*Promicromonospora sukumoe*	rhizosphere soil	solubilizes phosphate, mobilizes potassium, and withstands saline–alkaline stress	[96]
	*Agromyces*	*Agromyces humatus*	rhizosphere soil	mobilizes potassium and tolerates drought	[96]
	*Psychrobacillus*	*Psychrobacillus psychrodurans*	rhizosphere soil	mobilizes potassium, tolerates cold, and tolerates drought	[96]
	*Microbacterium*	*Microbacterium foliorum*	rhizosphere soil	solubilizes phosphate and promotes growth	[96]
	*Pseudomonas*	*Pseudomonas syringae*	rhizosphere soil	solubilizes phosphate and produces siderophores	[96]
	*Stutzerimonas*	*Stutzerimonas stutzeri*	rhizosphere soil	produces siderophores and tolerates saline–alkaline stress	[96]
	*Rhizobium*	*Rhizobium yanglingense*	nodule	enhances plant biomass and increases nitrogenase activity	[100]
	*Mesorhizobium*	*Mesorhizobium gobiense*	nodule	enhances plant biomass and increases nitrogenase activity	[100]
	*Pararhizobium*	*Pararhizobium herbae*	nodule	enhances plant biomass and increases nitrogenase activity	[100]

## Data Availability

No new data were created or analyzed in this study. Data sharing is not applicable to this article.
